# 

**Published:** 1917-06-16

**Authors:** 


### CONTENTS.

| | PAGE |
|---|---|
| **Report of the Highlands and Islands Medical Service Board** ... ... ... ... ... | 197 |
| **Baby Week** ... ... ... ... ... ... | 198 |
| **Hospital and Institutional News** ... ... ... | 199 |
| Our Hospital Sunday Number—Sir Alfred Keogh and Special Hospitals—American Surgeons for Work in England—The Victimisation of a Generous Profession—A Hospital for Those who have Lost Limbs in the War—The Hayes Gift to Chester Infirmary—An Irish Hospital Historian—The Belfast Eye Hospitals—"A Growing Grumble" in Swansea—Her Native City—River Trips for the Wounded—St. Dunstan's Hostel for Blinded Soldiers—Hospital Supplies of Wines and Spirits—A New Red Cross Hospital at Monmouth—The Value of Small Hospitals—A Hospital's Friends—The Old Site of Newcastle Infirmary—Panel Patients Without a Doctor—The Newest War Hospital—The Diminution of Panel Doctors in Birmingham—A Crèche Ward in Manchester—The Need for Day Nurseries—Dirty Workers—The Risk of Infection from Dental Instruments—The Dearth of Dental Surgeons—The late Dr. Bezly Thorne—Removal of the Board of Education—The Dearth of Recruits for the V.A.D.—Cycles for Doctors and Nurses—Poor-Law Visitors—Mental Hospital Attendants and the Tribunals—The Ex-Patient and Hospital Gazettes—This Week's Drug Market. | |
| **Industrial Efficiency and Fatigue** ... ... | 205 |
| Overwork and Overstrain. | |
| **Centralisation versus Decentralisation** ... | 206 |
| How to Fight Tuberculosis. | |
| **Recuperative Hostels**... ... ... ... ... | 207 |
| Has the Law Been Violated? | |
| **National Insurance Act Developments**... ... | 207 |
| The Revised Pension Arrangements, and a New Act. | |
| **The Editor's Letter-Box** ... ... ... ... | 209 |
| Recuperative Hostels—The Fruits of The Hospital's Teaching—A Fall in Infant Mortality. | |
| **Hospital and Institutional Results** ... ... | 210 |
| **The Matrons' and Sisters' Department** ... | 211 |
| Poor-Law Nursing in England: I. Sixty Years of its History. | |
| Round the Hospitals. | |
| American Nursing Opinion Ill-informed—Prejudice, Fiction, and Fact—A Travesty of the College Policy—Awkward Questions for State Registration Society—Miss Amy Hughes' Successor—The Irish Nurses' Association. | |
| **Food in Wartime** ... ... ... ... ... | 214 |
| The Foodstuffs Advisory Board Specimen Menu. | |
| **The Recreation Rooms** ... ... ... ... | 215 |
| Nigger Minstrels in a War Hospital. | |
| **Matrons' and Sisters' Appointments** ... ... | iv |
| **Parliament and the Profession** ... ... ... | iv |
| **Passing Events and Topics**... ... ... ... | iv |
| **The Institutional Worker** ... ... (Suppl.) | 1 |

